# Supplementary material for: Efficacy of vaccines based on chimeric or multiepitope antigens for protection against visceral leishmaniasis: A systematic review
Source: PLoS Negl Trop Dis. 2024 Dec 31;18(12):e0012757. doi: 10.1371/journal.pntd.0012757 (PMC11753665; doi:10.1371/journal.pntd.0012757)
Supplement: S1 File — (DOCX) [file pntd.0012757.s001.docx]

**S1 File.** Search strategy used in each database.

| **Data base** | **Search strategy** | **File** |
| --- | --- | --- |
| MEDLINE  (by Pubmed) | **Population:**  **#1** ((((((((((((((Leishmaniasis, Visceral[MeSH Terms]) OR (Leishmaniasis, Visceral[Title/Abstract])) OR (Kala Azar[Title/Abstract])) OR (Kala-Azar[Title/Abstract])) OR (Leishmania infantum[MeSH Terms])) OR (Leishmania infantum[Title/Abstract])) OR (Leishmania chagasi[Title/Abstract])) OR (Leishmania infantum chagasi[Title/Abstract])) OR (Leishmania (Leishmania) infantum[Title/Abstract])) OR (Leishmania (Leishmania) chagasi[Title/Abstract]))) OR (Leishmania donovani[MeSH Terms])) OR (Leishmania donovani[Title/Abstract])) OR (Leishmania (Leishmania) donovani[Title/Abstract])) | 1.211 |
|  | **Intervention:**  **#2** (((((((((((((((((((((Vaccines[MeSH Terms]) OR (Vaccines[Title/Abstract])) OR (Vaccine[Title/Abstract])) OR (Protozoan Vaccines[MeSH Terms])) OR (Protozoan Vaccines[Title/Abstract])) ) OR (Vaccines, Protozoan[Title/Abstract])) OR (Leishmaniasis Vaccines[MeSH Terms])) OR (Leishmaniasis Vaccines[Title/Abstract])) OR (Vaccines, Leishmaniasis[Title/Abstract])) OR (Leishmania Vaccines[Title/Abstract])) OR (Vaccines, Leishmania[Title/Abstract])) OR (Leishmania Vaccine[Title/Abstract])) OR (Vaccine, Leishmania[Title/Abstract])) OR (Leishmaniasis Vaccine[Title/Abstract])) OR (Vaccine, Leishmaniasis[Title/Abstract])) OR (Vaccination[MeSH Terms])) OR (Vaccination[Title/Abstract])) OR (Vaccinations[Title/Abstract])) OR (Active Immunization[Title/Abstract])) OR (Active Immunizations[Title/Abstract])) |  |
|  | #1 AND #2 |  |
| EMBASE | **Population:**  **#1** ('visceral leishmaniasis'/exp OR 'kala azar' OR 'kala-azar' OR 'kala-azar leishmaniasis' OR 'leishmaniasis visceralis' OR 'leishmaniasis, visceral' OR 'post-kala-azar dermal leishmaniasis' OR 'visceral leishmaniasis' OR 'leishmania infantum'/exp OR 'leishmania donovani infantum' OR 'leishmania infantum' OR 'leishmania donovani'/exp OR 'leishmania donovani') | 1.762 |
|  | **Intervention:**  **#2** ('vaccine'/exp OR 'combined vaccine' OR 'vaccin' OR 'vaccine' OR 'vaccine control' OR 'vaccine efficacy' OR 'vaccine potency' OR 'vaccine safety' OR 'vaccines' OR 'vaccines, combined' OR 'protozoal vaccine'/exp OR 'protozoal vaccine' OR 'protozoan vaccine' OR 'protozoan vaccines' OR 'leishmania vaccine'/exp OR 'leishmania vaccine' OR 'leishmania vaccines' OR 'canileish' OR 'leishmaniasis vaccine' OR 'leishmaniasis vaccines' OR 'leishvacin' OR 'letifend' OR 'neoleish' OR 'vaccination'/exp OR 'vaccination' OR 'vaccination policy' OR 'vaccination program' OR 'vaccination programme' OR 'vaccinotherapy') |  |
|  | #1 AND #2 AND ([embase]/lim OR [pubmed-not-medline]/lim) |  |
| Cochrane Library | **Population:**  **#1** MeSH descriptor: [Leishmaniasis, Visceral] explode all trees  **#2** MeSH descriptor: [Leishmania infantum] explode all trees  **#3** MeSH descriptor: [Leishmania donovani] explode all trees | 11 |
|  | **Intervention:**  **#4** MeSH descriptor: [Vaccines] explode all trees  **#5** MeSH descriptor: [Protozoan Vaccines] explode all trees  **#6** MeSH descriptor: [Leishmaniasis Vaccines] explode all trees  **#7** MeSH descriptor: [Vaccines] explode all trees |  |
|  | (#1 OR #2 OR #3) AND (#4 OR #5 OR #6 OR #7) |  |
| BVS | **Population:**  **#1** ((mh:(Leishmaniose viscérale)) OR (mh:(Leishmaniose Visceral)) OR (mh:(leishmaniasis visceral)) OR (Leishmaniose viscérale) OR (Leishmaniose Visceral) OR (leishmaniasis visceral) OR (Calazar) OR (mh:(Leishmania infantum)) OR (Leishmania infantum) OR (Leishmania donovani chagasi) OR (Leishmania donovani infantum) OR (Leishmania (Leishmania) infantum) OR (Leishmania (Leishmania) chagasi) OR (Leishmania chagasi) OR (Leishmania infantum chagasi) OR (mh:(Leishmania donovani)) OR (Leishmania donovani) OR (Leishmania (Leishmania) donovani)) | 107 |
|  | **Intervention:**  **#2** ((mh:(Vacinas )) OR (mh:(Vaccines )) OR (mh:(Vacunas)) OR (Vacinas ) OR (Vaccines ) OR (Vacunas) OR (Vacina) OR (mh:(Vacinas Protozoárias)) OR (mh:(Protozoan Vaccines)) OR (mh:(Vacunas Antiprotozoos)) OR (Vacinas Protozoárias) OR (Protozoan Vaccines) OR (Vacunas Antiprotozoos) OR (mh:(Vacinas contra Leishmaniose)) OR (mh:(Vaccins antileishmaniose)) OR (mh:(Vacunas contra la Leishmaniasis)) OR (Vacinas contra Leishmaniose) OR (Vaccins antileishmaniose) OR (Vacunas contra la Leishmaniasis) OR (Vacina contra Leishmania) OR (Vacina anti-Leishmania) OR (Vacina anti-Leishmaniose) OR (Vacina antiLeishmania) OR (Vacina antileishmaniose) OR (Vacina contra Leishmaniose) OR (Vacinas Antileishmaniose) OR (mh:(Vacinação )) OR (mh:(Vaccination )) OR (mh:(Vacunación)) OR (Vacinação ) OR (Vaccination ) OR (Vacunación) OR (Imunização Ativa)) |  |
|  | Bases available after withdrawing MEDLINE in the filter:  **#3** ( db:("LILACS" OR "colecionaSUS" OR "PAHOIRIS" OR "VETINDEX" OR "SES-SP" OR "WHOLIS" OR "WPRIM" OR "campusvirtualsp_brasil" OR "CUMED" OR "HISA" OR "IBECS" OR "MINSAPERU" OR "PREPRINT-FIOCRUZ" OR "PREPRINT-SCIELO" OR "SOF")) |  |
|  | #1 AND #2 AND #3 |  |
| **Total** | | **3.091** |
