# Supplementary material for: Efficacy of vaccines based on chimeric or multiepitope antigens for protection against visceral leishmaniasis: A systematic review
Source: PLoS Negl Trop Dis. 2024 Dec 31;18(12):e0012757. doi: 10.1371/journal.pntd.0012757 (PMC11753665; doi:10.1371/journal.pntd.0012757)
Supplement: Attachment — Submitted filename: Response to reviewers.docx [file pntd.0012757.s002.docx]

Dear Dr. Bradford S. McGwire and Dr. Susan Madison-Antenucci,

We would like to express our sincere gratitude for considering our manuscript titled "Efficacy of Chimeric or Multiepitope Antigen-Based Vaccines for Protection Against Visceral Leishmaniasis: A Systematic Review" for publication in PLOS Neglected Tropical Diseases. We greatly appreciate the time and effort invested by you and the reviewers in evaluating our submission.

We have thoroughly addressed all the comments and suggestions provided and have made the necessary revisions to enhance the quality of the manuscript. A summary of the changes, along with specific responses to each of the reviewers’ concerns, can be found below. These revisions are clearly highlighted in the updated manuscript for your convenience.

**Reviewer #1: The study presents well-articulated objectives, with a clear testable hypothesis, centered on the efficacy of vaccines based on chimeric or multiepitopic antigens for the protection against visceral leishmaniasis. The study design is adequate, using a systematic review of preclinical and clinical studies, with animal models such as rats, dogs and hamsters, which are appropriate for the hypothesis tested. The sample size seems sufficient in each individual study, although the limitation of some studies with small groups of animals may affect the generalizability of the results. Correct statistical analysis methods, such as limiting dilution and quantitative PCR, were used to support the conclusions, although the lack of details in some studies generated uncertain risk of bias. Regarding ethical requirements, the study mentions the use of the SYRCLE tool to assess the risk of bias in preclinical studies, suggesting the consideration of ethical guidelines, but without extensively detailing specific approvals for each study.**

Thank you for your thoughtful comments. The SYRCLE tool was utilized in this systematic review to assess the risk of bias in the included studies. Although this tool does not specifically address ethical aspects, we conducted an additional analysis focusing on the ethical approvals reported by the studies. We found that nearly all studies explicitly mentioned ethical approval by the relevant committees, except for two studies (Parody et al., 2004 and Coler et al., 2007). These exceptions have been highlighted in the manuscript to provide a comprehensive assessment. Please refer to lines 194–196 for further details.

**Reviewer #2: -In the collection of the characteristics of the included antigens, it is important to also collect data on from which species the original protein antigen was discovered. I.e. for the Coler et al 2007 study, it is provided as L. major stress-inducible protein (LmSTI1), but the species the antigen is discovered in was not mentioned for the other proteins. This could be important in the efficacy of a vaccine for L. infantum.**

Thank you for your suggestions. Information regarding the species from which the protein antigens were originally identified has been added to Table 2.

**-What were the predicted MHC haplotypes for the epitopes, and what is the MHC background of the animals tested in? Do these match? This may be good information to capture as well, as they may influence vaccine efficacy.**

Thank you for your suggestions. This information was added on Table 2

**- Do you think that including only studies assessing parasite load or positivity rate in studies involving natural infection will bias towards animal model research? Spleen parasite load assessment is a risky procedure in humans, and longitudinal natural infection assessments are often costly and therefore not performed a lot.**

The inclusion criterion based on these specific outcomes—studies evaluating parasite load or positivity rates in natural infection models was defined to align with our objective of identifying studies that best reflect vaccine efficacy. We believe that including studies focusing solely on immune responses (e.g., humoral or cellular) would not provide sufficiently robust predictors of vaccine effectiveness. While we anticipated identifying preclinical studies evaluating parasite load and clinical studies assessing positivity rates following natural infection, no human clinical studies meeting these criteria were found. This outcome likely reflects the limited number of vaccines that have progressed to human clinical trials. We recognize the important contribution of CHAKRAVARTY (2011), one of the most advanced human clinical studies on a multi-antigen protein vaccine. This study demonstrated the vaccine’s safety and its ability to induce T-cell cytokine production. If this vaccine were to advance to phase III clinical trials with protection evaluations, it would meet our inclusion criteria.

**Results**

**Reviewer #1: The analysis presented in the study is in line with the analysis plan described, following a systematic approach to evaluate the efficacy of vaccines based on chimeric and multiepitopic antigens against visceral leishmaniasis. The results are presented clearly and completely, with a detailed description of the reductions in parasite load in different animal models. The figures and tables used are of good quality and contribute to the clarity of the data, facilitating the understanding of the results. The information is well organized, offering an accurate view of the reviewed studies, with numerical data and percentages that support the conclusions of the study.**

**Reviewer #2: The analysis matches the analysis plan, and the tables are clear. I have no major comment on the results aside from this. Perhaps my comments on the Methods are also part of my comments on the Results since it is a systemic review.
A minor comment: what does 'Not made' mean in the tables? Undisclosed? Please clarify.**

Thank you for your comment. In Table 2, 'Not provided - NI' indicates that the original article did not include any information on the specific item. Conversely, 'Not made,' as used in the column referring to epitope prediction, means this analysis was not conducted in the studies. To ensure clarity, we have updated the caption for Table 2 to explain these terms explicitly.

**Conclusions**

**Reviewer #1: The conclusions of the study are well supported by the data presented, highlighting the potential of vaccines based on chimeric and multiepitopic antigens for protection against visceral leishmaniasis. The authors clearly discuss the limitations of the analysis, such as the risk of bias in some studies and the scarcity of clinical trials. In addition, they explore how the results can advance the development of new vaccines, highlighting the importance of additional studies to overcome the identified gaps. The public health relevance is addressed, emphasizing the need for effective vaccines to control visceral leishmaniasis, a disease that affects vulnerable populations in several regions of the world.**

**Reviewer #2: (No Response)**

**Editorial and Data Presentation Modifications?**

**Reviewer #1: No editorial or data presentation changes are necessary.**

**Reviewer #2: -L200: 'one antigen consisting of a pool of peptides selected through epitope prediction analyses.' is not clear to me. What is meant with this? Please clarify.**

Thank you for this opportunity to clear up this point. Athanasiou et al. (2017), used three synthetic multi-epitope peptides designed with selected epitopes from Cysteine Peptidase A (CPA), Histone H1 and Kinetoplast Membrane Protein 11 (KMP11). The multi-epitope peptides were then encapsulated together with MPLA molecules in Poly(lactic-co-glycolic acid) (PLGA) nanoparticles.

**-L233-L234: It is perhaps interesting to assess what the most potent adjuvant was?**

While determining the most potent adjuvant is certainly of interest, it is important to note that the effectiveness of an adjuvant depends on various factors, including the specific vaccine formulation, the type of immune response required, and the characteristics of the pathogen and disease stage. Based on the studies reviewed in our systematic analysis, saponin emerged as one of the most frequently used and promising adjuvants in combination with multiepitope antigens. Additionally, other adjuvants, such as MPLA and GLA-SE, also showed consistent potential in enhancing immune responses. However, due to the diversity in antigens tested across these studies, it is difficult to conclusively determine a single adjuvant as the most potent. This complexity is further discussed in lines 389-399 of the discussion.

**-L66 has a typo, should be 'severe' not 'sever'.**

The term has been rewritten. Please, see line 66.

**-L68 has a typo, should be 'caused' not 'cause'.**

The word has been rewritten. Please, see line 69.

**-L156 has a typo, should be 'tool' not 'toll'**

The term has been rewritten. Please, see line 160.

**-L386-L387: With 'less efficient in terms of hepatic parasite load', does this mean it decreases or increases the hepatic parasite load? Consider to clarify.**

Thank you for pointing that out. 'Less efficient in terms of hepatic parasite load' was intended to mean a lower hepatic parasite load. The sentence has been rewritten to make this clearer. Please, see lines 406-407.

**-L388: Consider to rewrite this in a 'negative' sense. So instead of 'Intravenous inoculation consistently results in effective infection', rather: 'Intravenous inoculation consistently fails to be protective' or something similar.**

Thank you for your comment. The sense of the phrase was to highlight that intravenous inoculation consistently leads to effective infection, which is critical for understanding the parasitic load dynamics in the context of our study. This information is particularly relevant for vaccine research, as it indicates that intravenous inoculation can result in higher parasite burdens in the spleen and liver of the animals. We believe the original wording effectively conveys this critical aspect and its significance.

**-What does 'Not made' mean in the tables? Undisclosed? Please clarify.**

In Table 2, 'Not made,' as used in the column referring to epitope prediction, indicates that this analysis was not performed in the studies. This clarification has now been included in the table subtitle for better understanding.

**Reviewer #1: The study presents a detailed and relevant systematic review on the efficacy of vaccines based on chimeric and multiepitopic antigens for protection against visceral leishmaniasis, a topic of high public health relevance, especially in endemic areas. Among the strengths, the scope of the research and the inclusion of data from preclinical and clinical studies stand out, which provides a broad view of the potential of these vaccines. In my opinion, the publication of the study is recommended.**

**Reviewer #2: To my knowledge, this is one of the first systematic reviews specifically focused on the efficacy of multi-epitope and chimeric vaccine antigens for visceral leishmaniasis. In my opinion, that makes it novel and interesting enough for publication. I particularly like that they aimed to not compare studies directly, but rather use the presented information to inform the public to stimulate rational development of new research into chimeric of multiepitope vaccines. However, there are some minor suggestions the manuscript could benefit from.**

**Major comments:**

**- On L82 to L84 it is written that incorporating multiepitope vaccine can increase the possibility of interaction with different mammalian MHC polymorphisms. However, compared to what do you mean? A live-attenuated or 'whole parasite' vaccine supposedly has all antigens of the parasite, so would theoretically have more possible epitopes that different MHC molecules could interact with. Similarly, a whole antigen-vaccine also has a lot of theoretical epitopes that can cover a large MHC space. Can you expand a little bit upon what you compare to and what the mechanism would be?**

Thank you for your thoughtful question. In the statement, the comparison was primarily intended to highlight the advantage of multiepitope vaccines over recombinant protein vaccines that often include a single antigen. While it is true that live-attenuated or whole-parasite vaccines theoretically present a broader range of epitopes, these approaches come with increased complexity and potential safety risks, such as the possibility of reversion to virulence or unintended immune responses. Multiepitope vaccines are designed to strategically select and combine highly immunogenic epitopes that are conserved and capable of interacting with a broader spectrum of MHC polymorphisms across diverse mammalian hosts. This targeted approach aims to optimize immune recognition and response without the safety concerns associated with live-attenuated or whole-parasite formulations. We have revised the text to clarify this distinction and further elaborate on the underlying mechanism (Lines 82-88)

**-On L367-L370 it is stated that the in silico prediction method has proven highly accurate in mapping immunogenic epitopes of Leishmania - I tend to disagree. These are methods that predict MHC epitopes or B cell epitopes, they do NOT predict immunogenicity. To my knowledge, there have been no validations of natural presentation of predicted MHC epitopes as of yet. While some predicted peptides may turn out 'immunogenic' in in vitro PBMC stimulations, this has not been proven to correlate with in vivo immunogenicity and protection. In fact, it has been observed in a recent cancer paper that in vitro PBMC stimulations with predicted peptides do not correspond to in vivo standards, because if the epitope is not naturally presented, there is no actual target the cognate T cells can recognize to elicit the immune response (https://doi.org/10.1016/j.jcyt.2023.12.002). I would consider to alter or remove this statement, although I welcome an open discussion on this as well if the authors want to keep it in.**

We appreciate your comment and the opportunity to clarify this point. We agree that in silico prediction tools focus on identifying epitopes with potential binding affinity to MHC molecules or interaction with B cells, which provides important insights into epitopes with immunogenic potential. However, we also understand that the ability of these predicted epitopes to induce a robust and protective immune response in vivo requires experimental validation. We have adjusted the text to better reflect this limitation, emphasizing that in silico prediction identifies epitopes with immunogenic potential based on MHC binding affinity and likely B cell interaction. We note, however, that effective immunogenicity depends on several additional factors, such as natural epitope presentation, the host immune context, and binding affinity, aspects that need confirmation in in vivo experiments. Please, see lines 372–379.

**-I would like to see which adjuvants were most promising.**

The selection of the ideal adjuvant is a complex task, as it requires the consideration of several formulation-specific factors, such as the route of administration, the type of immune response needed to control the infection, the type of pathogen, the stage of the disease, as well as safety and economic aspects (https://doi.org/10.1038/s41392-023-01557-7). Based on the studies included in our systematic review, we can highlight that saponin has been widely used in combination with various multi-epitope antigens, showing promising results in several formulations. Other adjuvants, such as MPLA and GLA-SE, have also been consistently used and demonstrated potential benefits. However, the diversity of antigens used in these studies makes it difficult to draw definitive conclusions regarding the superiority of any specific adjuvant. This issue is discussed in more detail in lines 389-399 of the discussion.

**-I would like to see the authors form a conclusion on what they think could help guide the rational development of new vaccine research: which adjuvants, what vaccination route, etc? Just a small sentence in the discussion would be fine.**

Thank you for this valuable suggestion. We have incorporated a conclusion in the discussion section, at lines 463-469.

**-Why is this the Das et al. manuscript on the LEISHDNAVAX vaccine not included (https://doi.org/10.1126/scitranslmed.3008222)? As far as I know, they fit within the inclusion criteria and do not fall within the exclusion criteria?**

The manuscript by Das et al. on the LEISHDNAVAX vaccine was not included because it does not meet the predefined inclusion criteria. Specifically, our criteria focused on the inclusion of multiepitope or chimeric proteins that were either synthesized or recombinantly produced. To clarify this point further, we added an explanation in lines 116-117. As such, DNA vaccines, such as the one reported in the cited manuscript, were excluded from our review.
